# Deep Learning-Based Carotid Plaque Ultrasound Image Detection and Classification Study

**DOI:** 10.31083/j.rcm2512454

**Published:** 2024-12-24

**Authors:** Hongzhen Zhang, Feng Zhao

**Affiliations:** ^1^Precision Medicine Innovation Institute, Anhui University of Science and Technology, 232001 Huainan, Anhui, China; ^2^General Surgery Department, The First Hospital of Anhui University of Science & Technology (Huai Nan First People’s Hospital), 232002 Huainan, Anhui, China

**Keywords:** artificial intelligence, deep learning techniques, carotid plaque, vulnerability, ischemic stroke

## Abstract

**Background:**

This study aimed to develop and evaluate the detection and classification performance of different deep learning models on carotid plaque ultrasound images to achieve efficient and precise ultrasound screening for carotid atherosclerotic plaques.

**Methods:**

This study collected 5611 carotid ultrasound images from 3683 patients from four hospitals between September 17, 2020, and December 17, 2022. By cropping redundant information from the images and annotating them using professional physicians, the dataset was divided into a training set (3927 images) and a test set (1684 images). Four deep learning models, You Only Look Once Version 7 (YOLO V7) and Faster Region-Based Convolutional Neural Network (Faster RCNN) were employed for image detection and classification to distinguish between vulnerable and stable carotid plaques. Model performance was evaluated using accuracy, sensitivity, specificity, F1 score, and area under curve (AUC), with *p* < 0.05 indicating a statistically significant difference.

**Results:**

We constructed and compared deep learning models based on different network architectures. In the test set, the Faster RCNN (ResNet 50) model exhibited the best classification performance (accuracy (ACC) = 0.88, sensitivity (SEN) = 0.94, specificity (SPE) = 0.71, AUC = 0.91), significantly outperforming the other models. The results suggest that deep learning technology has significant potential for application in detecting and classifying carotid plaque ultrasound images.

**Conclusions:**

The Faster RCNN (ResNet 50) model demonstrated high accuracy and reliability in classifying carotid atherosclerotic plaques, with diagnostic capabilities approaching that of intermediate-level physicians. It has the potential to enhance the diagnostic abilities of primary-level ultrasound physicians and assist in formulating more effective strategies for preventing ischemic stroke.

## 1. Introduction

Stroke is one of the most common diseases with the highest disability and mortality rates [1, 2, 3]. The incidence of ischemic stroke accounts for about 80% of all strokes. Moreover, about 70–80% of surviving patients have varying degrees of limb movement disorders, which seriously affects their quality of life [4, 5]. Therefore, timely rehabilitation treatment and dedicated care are needed to prevent disability.

The fundamental pathological basis of cardio-cerebral vascular diseases is atherosclerosis. The carotid artery, which connects the heart and the head, is a primary source of blood supply to the brain and is one of the most susceptible sites for atherosclerosis in the human body. Should carotid plaques rupture, they could potentially lead to cerebral artery embolism, which in turn may trigger clinical events such as ischemic stroke. Therefore, screening for carotid atherosclerotic plaques is important in preventing cardiovascular incidents. Ischemic stroke caused by carotid atherosclerotic plaque is the most common cause of death after heart disease and cancer. Some studies have suggested that 80% of cerebral ischemic processes are caused by carotid atherosclerotic vulnerable plaques [6, 7, 8, 9, 10, 11].

With its affordability, portability, and safety, ultrasound imaging has emerged as the preferred method for clinically screening carotid atherosclerotic plaques. It reveals the anatomical structure and characteristics of the vascular plaques and provides indicators of blood flow velocity within the vessels and the degree of vascular stenosis. However, in conventional ultrasound examinations, the diagnostic skills and experience of sonographers and the image quality of the ultrasound equipment can all influence the diagnostic outcomes of plaques. Consequently, applying deep learning technology to assist in the ultrasound diagnosis of carotid atherosclerotic plaques can significantly enhance the efficiency and accuracy of clinical diagnoses, presenting a vital clinical application value.

Currently, the clinical assessment methods for carotid atherosclerotic lesions mainly include ultrasound (US), computed tomographic angiography (CTA), and magnetic resonance imaging (MRI) [12, 13, 14, 15, 16, 17, 18, 19, 20, 21]. However, US imaging is more effective in identifying atherosclerotic plaques than other imaging modalities due to its wide range of applications and high detection rate of vulnerable plaques. Therefore, ultrasound forms the primary test for screening vulnerable plaques in the carotid arteries [5, 22, 23]. China has about 200,000 ultrasonographers conducting 2 billion annual ultrasound examinations, indicating a severe shortage of 150,000 ultrasonographers. Additionally, the traditional ultrasound diagnostic process is time-consuming and requires ultrasonographers to have extensive clinical experience, limiting the screening of many carotid atherosclerotic plaques in people at high risk of developing stroke [24, 25, 26, 27, 28, 29, 30]. The shortage, the uneven skill level of physicians, and the variable quality of image acquisition limit the early detection of carotid plaque vulnerable plaques in people at high risk of ischemic stroke. Therefore, an efficient and accurate method is needed to solve these difficulties. The recent emergence of deep learning technology has improved carotid plaque research globally [30, 31].

This study proposed an optimization scheme and algorithms to obtain a fully automated carotid artery plaque detection and classification model to provide the basis for the auxiliary diagnosis and treatment of ischemic stroke. Deep learning-based artificial intelligence technology has a better application prospect on carotid plaque ultrasound images, thus providing diagnostic assistance for junior physicians and effectively relieving the workload of ultrasonographers.

## 2. Materials and Methods

### 2.1 Research Population

Inclusion criteria included: (1) patients aged ≥18 years; (2) outpatients or inpatients with carotid atherosclerotic plaques diagnosed via carotid artery color Doppler ultrasound; (3) patients who did not undergo carotid vascular surgical procedures; (4) patients without a history of severe cerebrovascular disease.

Exclusion criteria: (1) age <18 years; (2) history of cervical vascular surgeries such as carotid endarterectomy, vascular bypass surgery, or carotid stent angioplasty; (3) exclusion of individuals who have previously undergone carotid vascular surgery; (4) exclusion of cerebrovascular events including but not limited to cerebral hemorrhage, cerebral infarction, and severe neurological dysfunction resulting from there; (5) patients with carotid artery occlusion; (6) individuals with physical limitations that prevent cooperation for carotid ultrasound examination.

A total of 5611 carotid ultrasound images of 3683 carotid atherosclerosis patients were collected from the ultrasound departments of the Eighth People’s Hospital of Shanghai, Fengxian District Central Hospital of Shanghai, the Second People’s Hospital of Guangdong Province, and the People’s Hospital of Huainan City, Anhui Province between 17 September 2020 and 17 December 2022. Specifically, 2657, 2099, 455, and 401 carotid ultrasound images were obtained from 1827 patients in Shanghai Eighth People’s Hospital, 1285 patients in Shanghai Fengxian District Central Hospital, 289 patients in Guangdong Guangdong Yuebei Second People’s Hospital, and 282 patients in Anhui Huainan City People’s Hospital, respectively. Qualified and trained ultrasonographers scanned the images using an advanced color ultrasound diagnostic instrument before saving them in the Digital Imaging and Communications in Medicine (DICOM) format in the corresponding folders.

### 2.2 Instrumentation and Data Acquisition

**Fig. 1.**
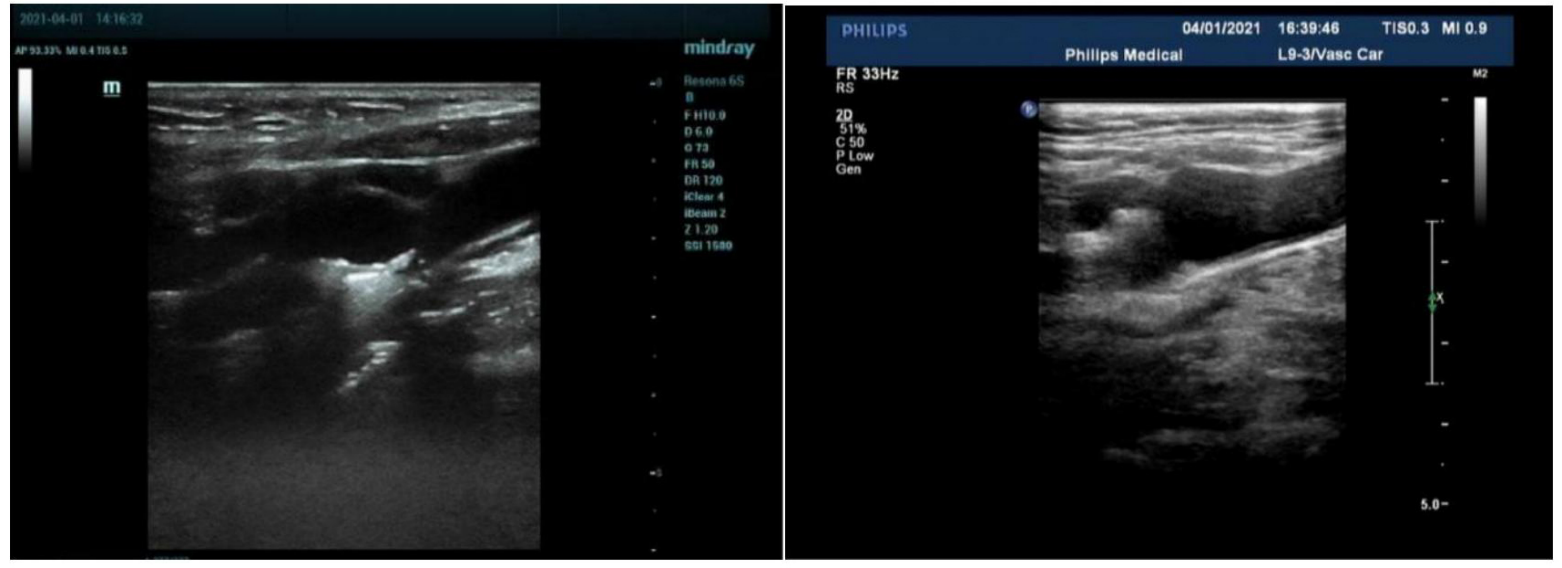
**Original ultrasound images of carotid plaque saved in DICOM format**. MI, mechanical index; 2D, two dimensional; FR, frequency; DICOM, digital imaging and communications in medicine; RS, radial strain.

Siemens S2000 (Model: 18L6 HD; Manufacturer: Siemens Healthineers; Origin: Erlangen, Germany); GE (Model: VIVID 7; Manufacturer: General Electric (GE) Healthcare; Origin: Waukesha, WI, USA); Esaote TWICE (Model: MyLab™Twice; Manufacturer: Esaote SpA; Origin: Turin, Italy); Esaote Class C (Model: MyLab Class C; Manufacturer: Esaote SpA; Origin: Turin, Italy); Hitachi A60 (Manufacturer: Hitachi, Ltd; Origin: Tokyo, Japan); Philips A70 (Model: Affiniti 70; Manufacturer: Koninklijke Philips N.V.; Origin: Anderhet, Netherlands); Philips Color Doppler (Model: EPIQ 7C; Manufacturer: Koninklijke Philips N.V.; Origin: Anderhet, Netherlands) ultrasound diagnostic machine, line array probe (frequency, 3–12 MHz) were used for data acquisition. Each image was acquired as follows: The patient was laid down with a pillow at the shoulder to expose the neck fully, and the head tilted backward and inclined to the opposite side. The patient’s bilateral carotid arteries were continuously swept from proximal to distal segments, and their transverse and longitudinal sections were observed following the Chinese Stroke Vascular Ultrasound Guidelines. The two dimensional (2D) morphology of the carotid arteries was observed dynamically. The carotid atherosclerotic plaques were detected and then placed in the center of the acquired images to fully display the observed plaque morphology, size, echogenicity, plaque integrity, and degree of vascular stenosis. Carotid plaque ultrasound images were stored in multiple sections and angles. The acquired carotid ultrasound images were saved in Digital Imaging and Communications in Medicine (DICOM) format (Fig. 1). Vulnerable plaques are defined as those with the following characteristics based on internationally recognized standards: A large lipid core, a thin fibrous cap, significant inflammatory response, and active neovascularization. We classified the plaques based on these features through imaging data [32, 33].

### 2.3 Data Splitting

The total dataset (5611 2D grey scale ultrasound images of carotid plaques) was divided into a training set (3927 images) and a test set (1684 images) in a ratio of 7:3. The dataset included 4135 images of vulnerable plaques and 1476 images of stable plaques.

**Fig. 2.**
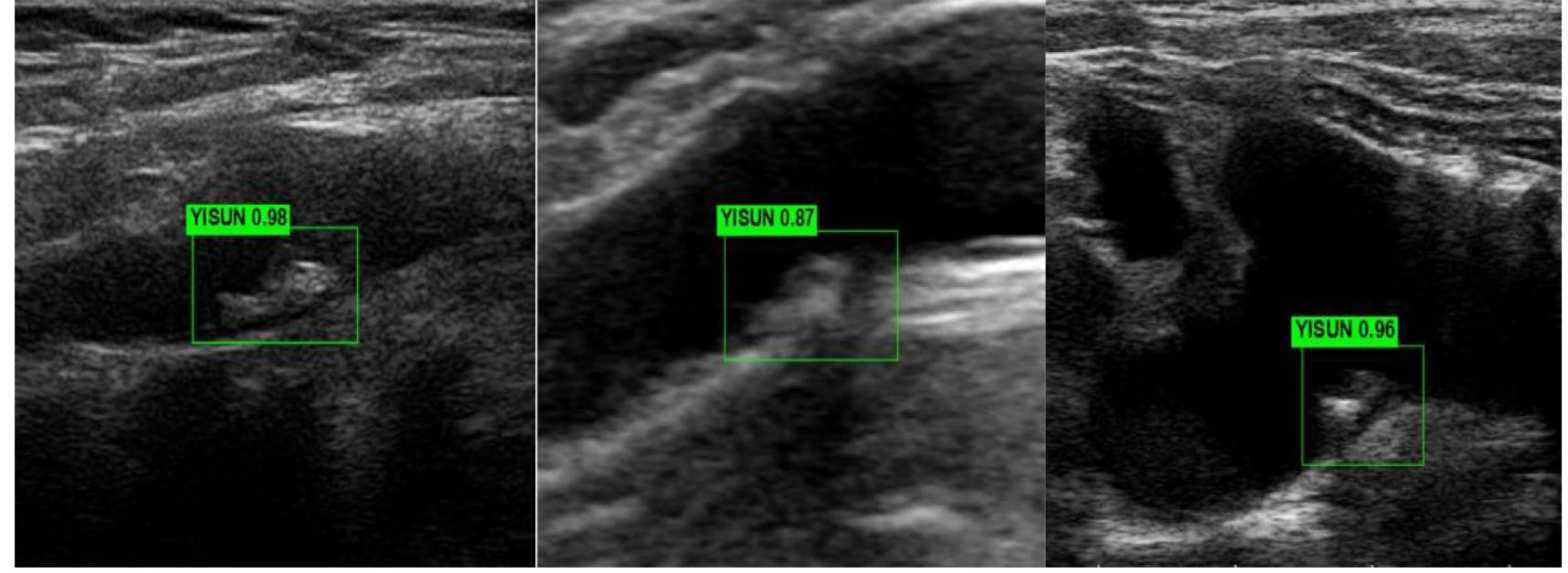
**Automatic detection and identification of vulnerable plaque ultrasound images**.

**Fig. 3.**
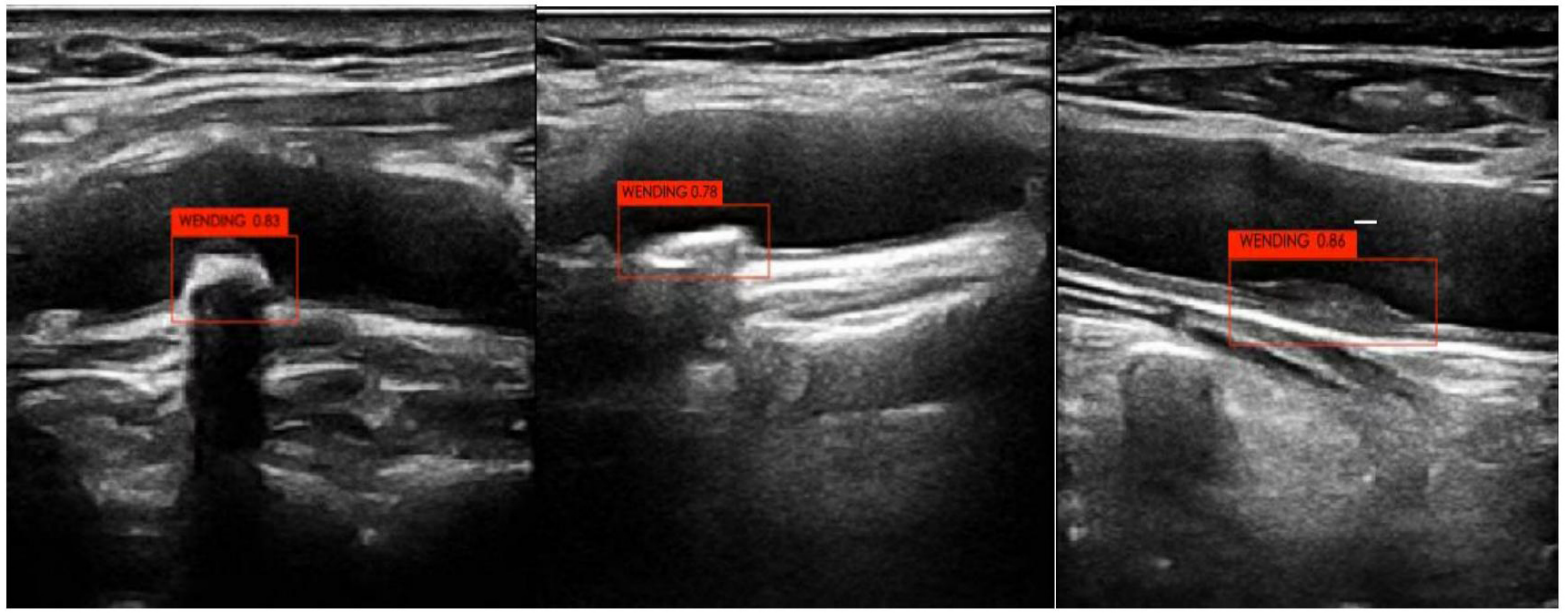
**Automatic detection and identification of stable plaques in ultrasound images**.

The neural network diagnosed the training and test sets separately after training. The carotid stable and vulnerable plaques were labeled “WENDIND” and “YISUN”, respectively. The deep learning model was used to interpret the carotid stable plaque/vulnerable plaque result as 1. The diagnostic output of each ultrasound image was between 0 and 1, where higher values indicated better diagnostic prediction (Figs. 2,3).

### 2.4 Indicators for Model Evaluation

In this study, accuracy (ACC), sensitivity (SEN), specificity (SPE), mean Intersection over Union (IoU), F1 score, receiver operating characteristic curve (ROC), and area under curve (AUC) were used to evaluate the performance of the model in the classification task. Accuracy measured the percentage of correctly classified samples in the test set (Eqn. 1), sensitivity measured the percentage of correctly classified positive samples (Eqn. 2), and specificity measured the percentage of correctly classified negative samples (Eqn. 3). These three metrics were calculated using true positive (TP), true negative (TN), false negative (FN), and false positive (FP).

TP is the number of positive samples with positive classification, i.e., the number of correctly identified carotid vulnerable plaque ultrasound images, while FP is the number of negative samples with positive classification, i.e., the number of vulnerable plaques identified as stable plaques. TN is defined as the number of negative samples with negative classification, i.e., the number of correctly identified stable plaques, while FN is the number of positive samples with negative classification, i.e., the number of incorrectly identified stable plaques.

Meanwhile, the AUC can be used to indicate the ability of the classifier to discriminate between samples and assess the performance of the classifier. AUC is used to weigh the performance of different classifiers between TP and FP error rates.

(1) $$\mathrm{ACC}=\frac{\mathrm{TP}+\mathrm{TN}}{\mathrm{TP}+\mathrm{TN}+\mathrm{FP}+\mathrm{FN}}\times 100\%$$

(2) $$\mathrm{SEN}=\frac{\mathrm{TP}}{\mathrm{TP}+\mathrm{FN}}\times 100\%$$

(3) $$\mathrm{SPE}=\frac{\mathrm{TN}}{\mathrm{FN}+\mathrm{FP}}\times 100\%$$

## 3. Results

### 3.1 Faster Region-Based Convolutional Neural Network (RCNN) Model Classification Performance

The SEN, SPE, ACC, and AUC of the Faster RCNN (ResNet 50) model for diagnosing carotid artery vulnerable plaque were 0.91, 0.69, 0.85, and 0.90, respectively, in the training set and 0.94, 0.71, 0.88, and 0.91, respectively, in the test set. The SEN, SPE, ACC, and AUC of the Faster RCNN (Inception V3) model for diagnosing carotid-vulnerable plaques were 0.89, 0.57, 0.79, and 0.86, respectively, in the training set and 0.91, 0.59, 0.83, and 0.85, respectively, in the test set. The performance indexes of the Faster RCNN (ResNet 50) model were significantly higher than those of the Faster RCNN (Inception V3) model (*p* < 0.05) (Table 1).

**Table 1.** **Comparison of Faster RCNN model confusion matrix and its parameter results**.

Evaluation indicators	Training set			Test set		
	Faster RCNN (ResNet 50)	Faster RCNN (Inception V3)	*p*-value	Faster RCNN (ResNet 50)	Faster RCNN (Inception V3)	*p*-value
Accuracy	0.85	0.79	<0.001	0.88	0.83	<0.001
Sensitivity	0.91	0.89	<0.001	0.94	0.91	<0.001
Specificity	0.69	0.57	<0.001	0.71	0.59	<0.001
AUC	0.90	0.86	<0.001	0.91	0.85	<0.001
Mean IoU	0.68	0.73	<0.001	0.72	0.74	<0.001
F1 score	0.91	0.89	<0.001	0.92	0.89	<0.001

Mean IoU, mean intersection over union; AUC, area under curve; RCNN, Region-Based Convolutional Neural Network.

**Fig. 4.**
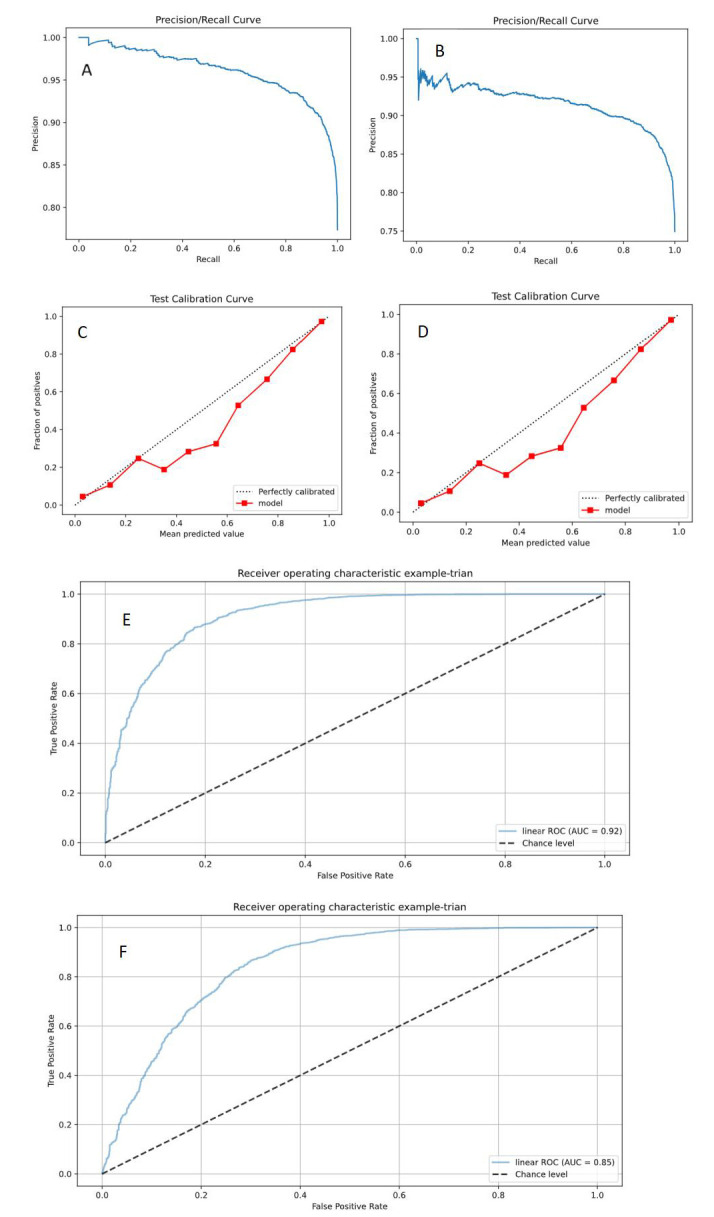
**Faster RCNN (ResNet 50, Inception V3) model detection results**. (A) PR curve of the Faster RCNN (ResNet 50) model; (B) PR curve of the Faster RCNN (Inception V3) model; (C) Calibration curve of the Faster RCNN (ResNet 50) model; (D) Calibration curve of the Faster RCNN (Inception V3) model; (E) ROC curve of the Faster RCNN (ResNet 50) model; (F) ROC curve of the Faster RCNN (Inception V3) model. PR, precision recall; ROC, receive operating characteristic; RCNN, Region-Based Convolutional Neural Network; AUC, area under curve.

However, the precision recall (PR) plot showed that the Faster RCNN (ResNet 50) model (PR = 0.956) outperformed the Faster RCNN (Inception V3) model (PR = 0.927). The Faster RCNN (ResNet 50) model had better detection classification performance in detecting plaque properties than the Faster RCNN (Inception V3) model (Fig. 4).

### 3.2 You Only Look Once Version 7 (YOLO V7) Model Classification Performance

The SEN, SPE, ACC, and AUC of YOLO V7 (ResNet 50) were 0.91, 0.69, 0.85, and 0.90, respectively, in the training set and 0.94, 0.71, 0.88, and 0.91, respectively, in the test set. The SEN, SPE, ACC, and AUC of YOLO V7

(Inception V3) were 0.89, 0.57, 0.79, and 0.86, respectively, in the training set, and 0.91, 0.59, 0.83, and 0.85, respectively, in the test set.

The performance indexes of the YOLO V7 (ResNet 50) model were significantly higher than those of the YOLO V7 (Inception V3) model (*p* < 0.05) (Table 2).

**Table 2.** **Comparison of the model confusion matrix and its parameter results for YOLO V7**.

Evaluation indicators	Training set			Test set		
	YOLO V7 (ResNet 50)	YOLO V7 (Inception V3)	*p*-value	YOLO V7 (ResNet 50)	YOLO V7 (Inception V3)	*p*-value
Accuracy	0.83	0.84	<0.001	0.86	0.83	<0.001
Sensitivity	0.91	0.90	<0.001	0.94	0.91	<0.001
Specificity	0.59	0.63	<0.001	0.61	0.60	<0.001
AUC	0.90	0.82	<0.001	0.86	0.83	<0.001
Mean IoU	0.68	0.68	<0.002	0.68	0.69	<0.001
F1 score	0.90	0.88	<0.001	0.91	0.89	<0.001

Mean IoU, mean intersection over union; AUC, area under curve; YOLO V7, You Only Look Once Version 7.

**Fig. 5.**
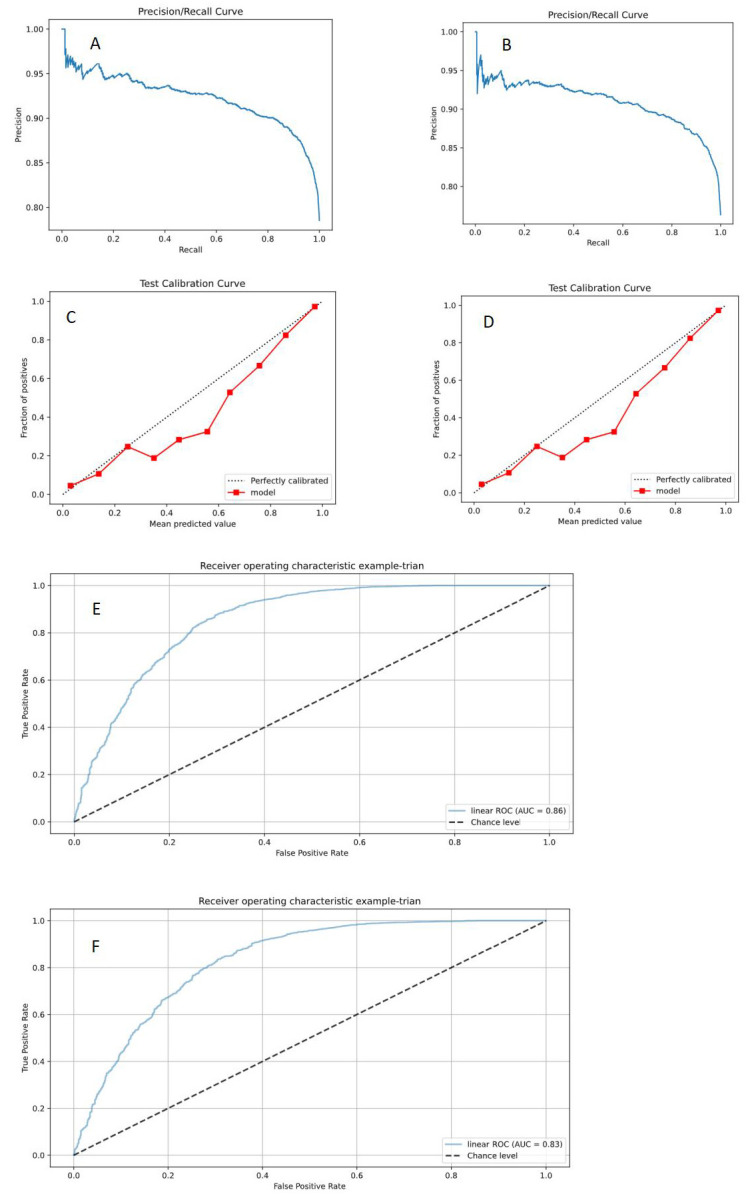
**YOLO V7 (ResNet 50, Inception V3) model detection results**. (A) PR curve of YOLO V7 (ResNet 50) model; (B) PR curve of YOLO V7 (Inception V3) model; (C) calibration curve of YOLO V7 (ResNet 50) model; (D) calibration curve of YOLO V7 (Inception V3) model; (E) ROC curve of YOLO V7 (ResNet 50) model; (F) ROC curve of the YOLO V7 (Inception V3) model. PR, precision recall; ROC, receive operating characteristic; YOLO V7, You Only Look Once Version 7; AUC, area under curve.

Although the PR plot indicated that the YOLO V7 (ResNet 50) model (PR = 0.936) had performed better than the YOLO V7 (Inception V3) model (PR = 0.927), the model calibration of the YOLO V7 (ResNet 50) model (mean intersection over union, Mean IoU = 0.68) was slightly lower than that of the YOLO V7 (Inception V3) (Mean IoU = 0.74). Notably, the YOLO V7 (Inception V3) model exhibited higher accuracy than the YOLO V7 (ResNet 50) model (Fig. 5).

These findings indicate that the YOLO V7 (ResNet 50) model has better detection and classification performance than the YOLO V7 (Inception V3) model.

### 3.3 Comparison of Classification Performance between Faster RCNN Model and YOLO V7 Model

The Faster RCNN (ResNet 50) model showed the best performance among the four models, with SEN, SPE, ACC, AUC, and F1 scores of 0.94, 0.71, 0.88, 0.91, and 0.92, respectively, in the test set. Similarly, the Faster RCNN (Inception V3) model showed the best performance among the four models based on calibration (Mean IoU = 0.74) with the highest model consistency. Therefore, the Faster RCNN (ResNet 50) model was optimal for diagnosing carotid vulnerable plaques among the four models, with an AUC of 0.91. In contrast, the YOLO V7 (Inception V3) model was the worst model for diagnosis, with an AUC of 0.83 (*p* < 0.05) (Table 3).

**Table 3.** ** Comparison of the confusion matrix results and its parameters for the four models in the test set**.

Models	Accuracy	Sensitivity	Specificity	AUC	Mean IoU	F1 score	*p*-value
Faster RCNN (ResNet 50)	0.88	0.94	0.71	0.91	0.72	0.92	<0.001
Faster RCNN (Inception V3)	0.83	0.91	0.59	0.85	0.74	0.89	<0.001
YOLO V7 (ResNet 50)	0.86	0.94	0.61	0.86	0.68	0.91	<0.001
YOLO V7 (Inception V3)	0.83	0.91	0.60	0.83	0.69	0.89	<0.001

Mean IoU, mean intersection over union; AUC, area under curve; YOLO V7, You Only Look Once Version 7; RCNN, Region-Based Convolutional Neural Network.

## 4. Discussion

Ultrasound images have become a popular research object in the medical field. However, in acquiring ultrasound images of carotid plaques, the quality of ultrasound images and the level of ultrasound image acquisition varies, seriously affecting the accurate classification and diagnosis of carotid plaques by sonographers. With the continuous development of computer technology and information science, scientific research database management is becoming more diversified, convenient, and networked. Carotid plaque ultrasound image data collected in this study were collected from Shanghai Eighth People’s Hospital, Shanghai Fengxian Central Hospital, Guangdong Province, North Guangdong Second People’s Hospital, Huainan People’s Hospital, Anhui Province respectively, and the inspection equipment used for image collection was seven kinds of ultrasound instruments of various brands and models at home and abroad. As we all know, for the training and verification of deep learning models, the larger the sample size, the better the performance of the selected models. In this study, we included 3683 patients with carotid atherosclerotic plaque replacement and rationally designed the carotid plaque ultrasound image data of the included subjects.

As deep learning technology gains prevalence in the medical domain, it has proven capable of autonomously adapting to the nonlinear characteristics of medical images through sophisticated multilayer mappings, thereby uncovering intrinsic features within the raw data. This confers an improved adaptability and generalization potential. Zhu *et al*. [20] demonstrated that deep learning can exploit a richer array of image data information compared to traditional manual feature extraction, a benefit particularly pronounced in the context of carotid plaque image feature extraction. Furthermore, the feature extraction approach for carotid plaque images, developed by Skandha *et al*. [23] and based on deep learning, excels in addressing the classification complexities associated with different types of carotid plaque images, reinforcing identification capabilities and facilitating the extraction and integration of multidimensional features from carotid ultrasound images.

**Fig. 6.**
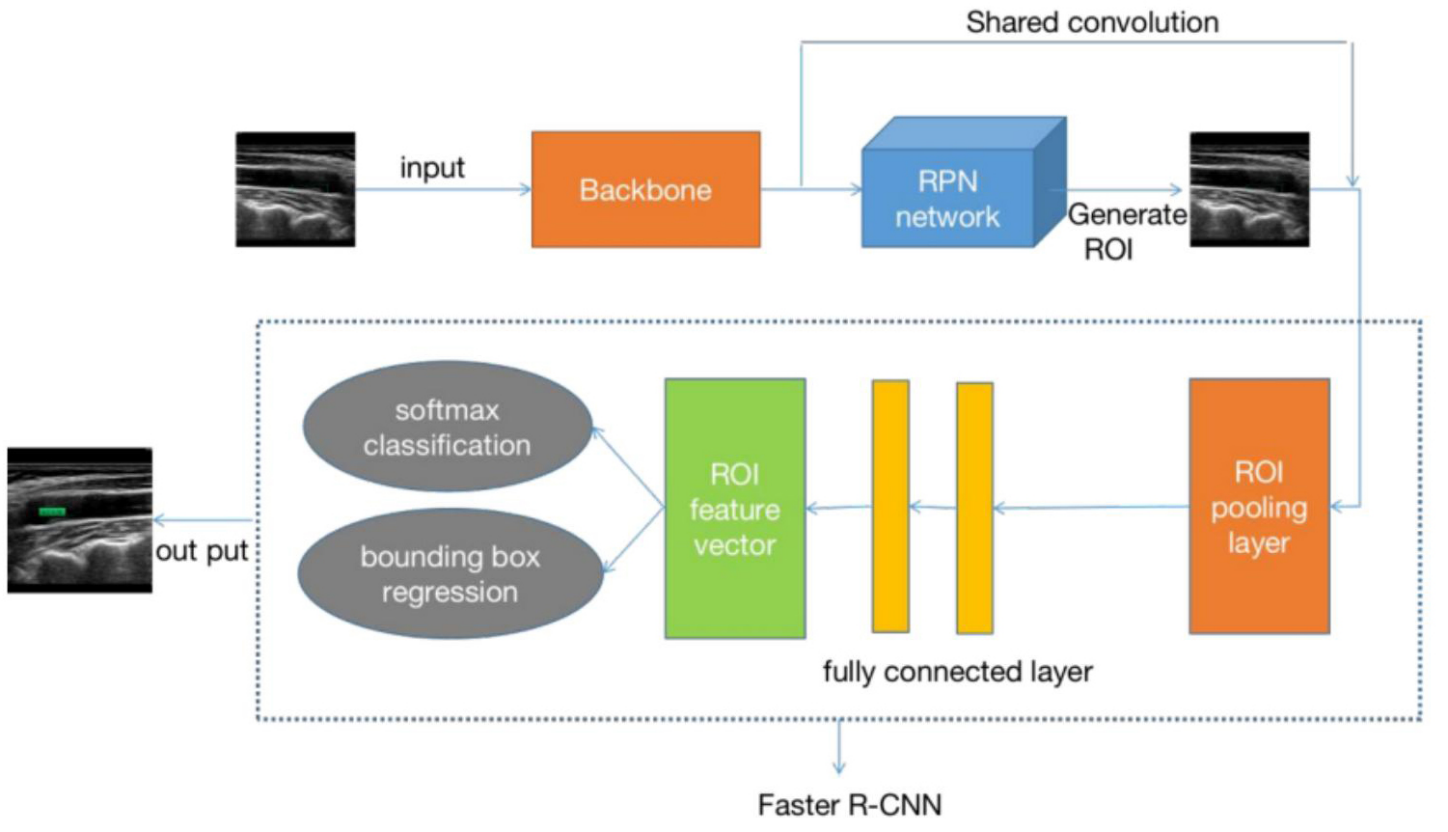
**Schematic of the Faster RCNN model**. RCNN, Region-Based Convolutional Neural Network; RPN, region proposal network; ROI, region of interest.

**Fig. 7.**
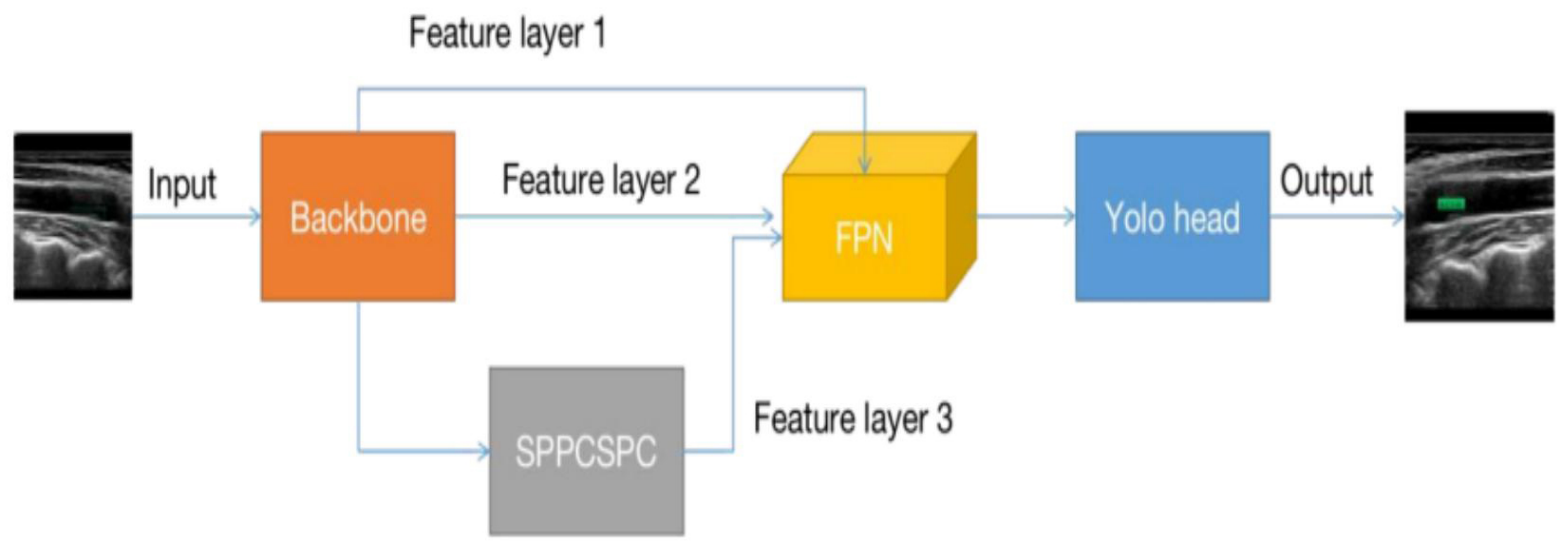
**Schematic diagram of the YOLO V7 model**. YOLO, You Only Look Once; FPN, feature pyramid network; SPPCSPC, spatial pyramid pool construction statistical process control.

While deep learning technology boasts many strengths, its challenges, and limitations include the substantial data requirements of artificial intelligence systems and the necessity to tailor deep learning algorithms to the unique characteristics of different diseases. Consequently, the flexible design of distinct deep learning models and algorithmic workflows for various carotid plaque types to achieve efficient and precise detection and classification represents a gap in the current research landscape. This study utilizes the Faster RCNN (Fig. 6) and YOLO V7 (Fig. 7) models as the cornerstone of deep learning convolutional neural networks, deploying two diverse feature extraction networks (Resnet 50 and Inception V3) to identify and classify carotid plaques from ultrasound images, distinguishing between vulnerable and stable plaques. The performance of four distinct deep-learning models in detecting and classifying carotid plaques is meticulously analyzed and discussed.

This study employs the method of artificial intelligence deep learning, utilizing ResNet 50 and Inception V3 as the fundamental feature extraction network models. It involves training the carotid plaque ultrasound images using the YOLO V7 and Faster RCNN models, respectively. The Faster RCNN model can achieve real-time detection while maintaining high accuracy, simultaneously handling object detection and classification, thereby reducing algorithmic complexity. This model also exhibits better performance in detecting small and dense targets. The YOLO V7 model features a lightweight network architecture, which consumes less computational resources and can be deployed on smaller devices. However, the YOLO V7 model has slightly lower detection accuracy than the Faster RCNN model and is less effective in detecting small and dense targets.

The subject of this study is carotid ultrasound images, with carotid plaques being considered as small target detection. These research results indicate that, compared to the YOLO V7 model based on two different feature extraction networks (ResNet 50 and Inception V3), the Faster RCNN (ResNet 50) model achieved the highest AUC of 0.91, while the Faster RCNN (Inception V3) model had an AUC of 0.85, slightly lower than the YOLO V7 (ResNet 50) model’s AUC of 0.86. The YOLO V7 (Inception V3) model performed the worst, with an AUC of 0.83. Therefore, in the context of this study, the Faster RCNN model demonstrates superior performance compared to the YOLO V7 model. In the analysis of medical ultrasound images, the uncertainty of the size and quantity of objects within the images leads to varying detection outcomes based on different feature extraction networks (ResNet 50 and Inception V3). Due to the complexity of ultrasound images, characterized by noise interference and low contrast, the ResNet 50 network possesses stronger image feature extraction capabilities than the Inception V3 network, allowing for more precise extraction of target object features. The Faster RCNN model, based on the ResNet 50 feature extraction network, exhibits robust feature extraction and recognition abilities.

This study delved deeply into the performance of various deep-learning models in detecting and classifying carotid plaque ultrasound images. Notably, interobserver and intraobserver variability are significant factors affecting ultrasound image interpretation accuracy. Therefore, a meticulous analysis of such variabilities is crucial for assessing the stability and reliability of the models. Interobserver variability generally stems from the differences in image feature recognition and interpretation among different physicians, while intraobserver variability is related to the consistency of interpretation by the same physician for the same image at various times. To evaluate the impact of these variabilities on our study results, we annotated the images in our dataset multiple times and calculated the coefficients of variation for interobserver and intraobserver variabilities. According to related research [34], interobserver variability in our study was kept within an acceptable range, indicating a high level of agreement among professional physicians in recognizing carotid plaque ultrasound images. However, intraobserver variability was relatively high, which might be due to the complexity and subjectivity of the ultrasound images. Nevertheless, the Faster RCNN (ResNet 50) model exhibited a stable performance in the test set, demonstrating good classification efficacy. Analysis of the interobserver and intraobserver variabilities showed that the Faster RCNN (ResNet 50) model had high stability in the accuracy, sensitivity, and specificity for carotid plaque classification, comparable to the diagnostic level of intermediate-level physicians. Specifically, the coefficients of variation for interobserver accuracy, sensitivity, and specificity were 0.05, 0.04, and 0.06, respectively, while the coefficients of variation for intraobserver were 0.03, 0.02, and 0.04, respectively [34, 35]. These results suggest that the Faster RCNN (ResNet 50) model significantly reduces interobserver and intraobserver variabilities, contributing to improved consistency in the classification of carotid plaque ultrasound images. Moreover, the high stability of the model also provides a reliable auxiliary tool for ultrasound physicians, aiding in enhancing their diagnostic capabilities and promoting the development of more effective strategies for preventing ischemic stroke.

The application of AI tomography in analyzing carotid plaque characteristics has provided a new perspective for a deeper understanding of plaque nature. Tomography technology can capture information on carotid plaques from multiple angles, aiding in the revelation of the plaque’s internal microstructure [36]. Combined with deep learning models, features of carotid plaques can be automatically extracted, offering a more comprehensive basis for clinical diagnosis [37]. The use of AI tomography in analyzing carotid plaque characteristics holds promise for providing more precise predictive indicators for the early identification of major vascular strokes.

In the current healthcare environment, the importance of early identification and accurate classification of carotid atherosclerotic plaques in preventing ischemic strokes must be addressed [38]. By comparing the performance of different deep learning models in the detection and classification of carotid plaque ultrasound images, this study has unveiled the immense potential of deep learning technology in the early identification of major vascular strokes. The YOLO V7 and Faster RCNN models demonstrated exceptional classification performance in this study, with the Faster RCNN (ResNet 50) model particularly highlighted for its high accuracy, sensitivity, and AUC values, indicating its potential in the early identification of carotid atherosclerotic plaques. These models can effectively identify vulnerable plaques, which is crucial for clinicians to develop intervention strategies and preventive measures. Early identification of carotid plaques not only aids in risk assessment but also guides clinicians in targeted therapeutic interventions, such as pharmacological treatment, lifestyle modifications, or surgical interventions. For instance, the diagnostic level of the Faster RCNN (ResNet 50) model is nearly equivalent to that of a mid-level physician, suggesting its potential to enhance the diagnostic capabilities of primary ultrasound physicians in primary healthcare settings, thus enabling early intervention in the stages of major vascular stroke onset. Furthermore, the rapid detection and classification abilities of deep learning models contribute to efficient and precise plaque identification in large-scale population screenings, which is of significant importance in public health. By identifying carotid atherosclerotic plaques early, we can provide timely treatment and intervention for patients, reducing the risk of ischemic strokes. Therefore, our findings provide robust evidence for applying deep learning technology in the early identification of major vascular strokes.

However, the current study does have certain limitations. Firstly, the dataset size is limited, which may result in insufficient generalization of the models. Future research could expand the dataset size to enhance model stability and accuracy. Secondly, this study focuses solely on detecting and classifying carotid plaques without addressing other factors that may contribute to major vascular strokes. In practical applications, it may be beneficial to incorporate other biomarkers and clinical indicators to improve predictive performance. Future research will further explore the applicability of these models in different populations and the methods through which they can be integrated into existing clinical practices to achieve optimal preventive and treatment strategies.

## 5. Conclusions

This study evaluated four deep-learning models for automatically detecting and classifying carotid atherosclerotic plaques in ultrasound images. The Faster RCNN (ResNet 50) model emerged as the most effective, demonstrating high accuracy and reliability. Thus, the Faster RCNN (ResNet 50) model holds promise for aiding primary physicians in identifying vulnerable plaques, enhancing diagnosis rates, and guiding personalized interventions for high-risk ischemic stroke patients.

## Availability of Data and Materials

The datasets used or analyzed during the present study are available from the corresponding author upon reasonable request.
